# Role of Arginase in Vessel Wall Remodeling

**DOI:** 10.3389/fimmu.2013.00111

**Published:** 2013-05-13

**Authors:** William Durante

**Affiliations:** ^1^Department of Medical Pharmacology and Physiology, University of Missouri-ColumbiaColumbia, MO, USA

**Keywords:** arginase, vascular remodeling, smooth muscle cell proliferation, endothelial dysfunction, nitric oxide

## Abstract

Arginase metabolizes the semi-essential amino acid l-arginine to l-ornithine and urea. There are two distinct isoforms of arginase, arginase I and II, which are encoded by separate genes and display differences in tissue distribution, subcellular localization, and molecular regulation. Blood vessels express both arginase I and II but their distribution appears to be cell-, vessel-, and species-specific. Both isoforms of arginase are induced by numerous pathologic stimuli and contribute to vascular cell dysfunction and vessel wall remodeling in several diseases. Clinical and experimental studies have documented increases in the expression and/or activity of arginase I or II in blood vessels following arterial injury and in pulmonary and arterial hypertension, aging, and atherosclerosis. Significantly, pharmacological inhibition or genetic ablation of arginase in animals ameliorates abnormalities in vascular cells and normalizes blood vessel architecture and function in all of these pathological states. The detrimental effect of arginase in vascular remodeling is attributable to its ability to stimulate vascular smooth muscle cell and endothelial cell proliferation, and collagen deposition by promoting the synthesis of polyamines and l-proline, respectively. In addition, arginase adversely impacts arterial remodeling by directing macrophages toward an inflammatory phenotype. Moreover, the proliferative, fibrotic, and inflammatory actions of arginase in the vasculature are further amplified by its capacity to inhibit nitric oxide (NO) synthesis by competing with NO synthase for substrate, l-arginine. Pharmacologic or molecular approaches targeting specific isoforms of arginase represent a promising strategy in treating obstructive fibroproliferative vascular disease.

## Introduction

Arterial remodeling is characterized by alterations in the structure and function of the vascular wall in response to specific pathophysiologic stimuli. Although vascular remodeling naturally occurs in response to aging, it also arises in response to injury and disease. The remodeling response is characterized by alterations of one or all three layers of the blood vessel wall: the adventitia, media, and intima. It is driven by numerous complex and interrelated pathological processes that influence both the cellular and non-cellular components of the vascular wall (Orford et al., 2000; Jeffrey and Wanstall, 2001; Dzau et al., 2002; van Varik et al., 2012). The proliferation of cells within the vessel wall is a major contributor to arterial remodeling. The proliferation of vascular smooth muscle cells (SMCs) and/or endothelial cells (ECs) leads to intimal thickening, hyperplasia, or hypertrophy of SMCs results in medial thickening, and fibroblast proliferation causes adventitial expansion. In addition, the recruitment of inflammatory cells from the circulation alters the cellular composition and mass within the vessel wall. These cellular modifications are often accompanied by increased deposition of extracellular matrix material, such as collagen and fibronectin, as well as the fragmentation and degradation of elastin which adversely affects the biomechanical properties of blood vessels. Furthermore, changes in cell phenotype supports vascular remodeling. The dedifferentiation of vascular SMCs from a contractile to a synthetic phenotype that occurs in response to arterial injury aids vascular remodeling by augmenting the secretion of collagen and other extracellular matrix proteins (Owens et al., 2004). Synthetic SMCs also generate matrix metalloproteinases that facilitates SMC migration from the media to the intima by detaching these cells from the basement membrane and extracellular matrix (Bendeck et al., 1996). Moreover, the phenotype of ECs plays a pivotal role in the remodeling response. In response to blood flow and shear stress, ECs release a myriad of humoral factors, including the gas nitric oxide (NO), which maintains vascular SMCs in a quiescent non-proliferative and differentiated non-secretory state (Bonetti et al., 2003; Versari et al., 2007). However, EC function decreases with age and disease resulting in diminished NO synthesis and enhanced production of cytokines and chemokines that triggers the recruitment and infiltration of immune cells into the vessel wall. Moreover, dysfunctional ECs generate various growth factors that stimulate SMCs to proliferate, dedifferentiate, and synthesize collagen. Finally, leukocytes that traffic into the vessel wall exhibit distinct phenotypes that can influence the structure of the vessel wall by enhancing or resolving vascular inflammation.

Arterial remodeling is a salient feature of aging and plays a fundamental role in the development of several vascular disorders, including atherosclerosis, restenosis after percutaneous coronary intervention, post-transplant vasculopathy, systemic and pulmonary hypertension, and aortic aneurysm and dissection (Ross, 1999; Jeffrey and Wanstall, 2001; Dzau et al., 2002; Schiffrin, 2012). The targeting of specific pathophysiological pathways that contribute to arterial remodeling offers a potential approach for therapeutic intervention. Despite extensive investigation, the discovery and translation of potential targets of vascular remodeling to the clinic has met with limited success. However, studies in the past few years have identified arginase as a promising therapeutic target that underlies many of the pathophysiological processes that contribute to arterial remodeling (Durante et al., 2001; Wei et al., 2001; Li et al., 2002; Chicoine et al., 2004; Ryoo et al., 2008; Kim et al., 2009; Chen et al., 2012; Ming et al., 2012; Cho et al., 2013). This article will review the mechanisms by which arginase promotes aberrant vascular remodeling in arterial injury, pulmonary and systemic hypertension, aging, and atherosclerosis, focusing on the cellular actions of enzyme. In addition, it will highlight arginase as a novel therapeutic modality in the prevention and treatment of occlusive vascular proliferative disease.

## l-Arginine Metabolism by Vascular Cells

**Figure 1:**
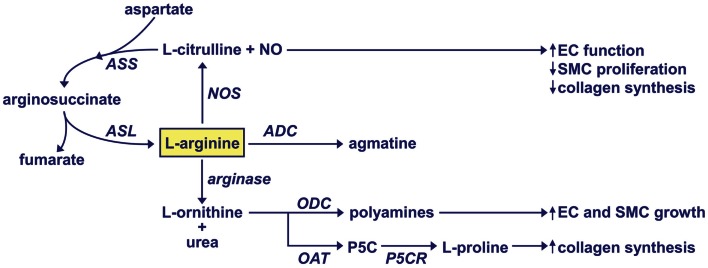
**Regulation of l-arginine metabolism by vascular cells**. NO, nitric oxide; NOS, nitric oxide synthase; ASS, argininosuccinate synthetase; ASL, argininosuccinate lyase; ADC, arginine decarboxylase; ODC, ornithine decarboxylase; OAT, ornithine aminotransferase; P5C, pyrroline-5-carboxylate; P5CR, pyrroline-5-carboxylate reductase.

l-Arginine is a semi-essential amino acid that is involved in numerous physiological processes. It is a necessary precursor for protein and creatinine biosynthesis and plays a role in modulating nitrogen balance. In addition, l-arginine is metabolized by vascular cells to a number of important regulatory molecules (Figure 1). Studies in the late 1980s, discovered that l-arginine is oxidized to NO and l-citrulline by nitric oxide synthase (NOS) (Hibbs et al., 1988; Palmer et al., 1988). l-Citrulline is subsequently recycled back to l-arginine by the successive actions of argininosuccinate synthetase and argininosuccinate lyase (Hecker et al., 1990). There are three distinct isoforms of NOS; neuronal NOS (nNOS or NOS-1), endothelial NOS (eNOS or NOS-3), and inducible NOS (iNOS or NOS-2) (Forstermann and Sessa, 2012). These isozymes display differences in tissue distribution, intracellular localization, molecular regulation, enzyme kinetics, and calcium-dependency. Aside from serving as a substrate, l-arginine plays an important structural and functional role by facilitating the intracellular assembly of the dimeric form of NOS and the proper coupling between the oxidative and reductive domains of the enzyme (Baek et al., 1993; Forstermann and Munzel, 2006). The release of NO by vascular cells plays an important homeostatic role in the circulation by inhibiting vascular tone, platelet aggregation, leukocyte adhesion and infiltration into the vessel wall, and the proliferation and migration of vascular SMCs (see Durante, 2001; Forstermann and Sessa, 2012).

Studies in the 1990s revealed that vascular cells also express the enzyme arginase that catalyzes the hydrolysis of l-arginine to l-ornithine and urea (Buga et al., 1996; Durante et al., 1997). There are two distinct isoforms of arginase, arginase I and II, which are encoded by separate genes and share approximately 60% amino acid sequence homology (Dizikes et al., 1986; Vockley et al., 1996). These isozymes exhibit differential tissue distribution, subcellular localization, and molecular regulation (Jenkinson et al., 1996). Arginase I is a cytosolic enzyme that is abundantly expressed in the liver and plays an essential role in hepatic urea cycle. In contrast, arginase II is a mitochondrial enzyme that is widely expressed outside the liver, most prominently in the kidney and prostate (Vockley et al., 1996; Morris et al., 1997). Notably, arginase I germline knockout mice die shortly after birth due to severe hyperammonemia whereas arginase II-deficient mice are viable (Shi et al., 2001; Iyer et al., 2002). The arginase product l-ornithine is further metabolized by the cytosolic enzyme ornithine decarboxylase to the polyamine putrescine which forms the successive polyamines, spermine, and spermidine (Tabor and Tabor, 1984). SMC and EC proliferation is preceded by increases in polyamine synthesis and inhibition of polyamine formation abolishes cell growth (Morrison and Seidel, 1995; Durante et al., 1996b, 1998). l-Ornithine is also catabolized by the mitochondrial enzyme ornithine aminotransferase to pyrroline-5-carboxylate, which is further metabolized to l-proline by the enzyme pyrroline 5-carboxylate reductase. l-Proline is required for the synthesis of many structural proteins, including collagen (Durante et al., 2000, 2001). Finally, l-arginine may also be metabolized by the enzyme arginine decarboxylase to agmatine, which elicits anti-proliferative effects (Regunathan et al., 1996). However, these latter findings need further corroboration.

## Regulation of Arginase Expression in Vascular Cells

Recent studies have documented the presence of arginase in a multitude of blood vessels, including the aorta, carotid and pulmonary artery, retinal arteries, coronary arteries, and gracilis muscle arterioles (see Durante et al., 2007; Morris, 2009; Elms et al., 2013). Arginase I and II have been detected in both vascular SMCs and ECs but the abundance of each isoform is variable and likely reflects differences between animal species, vascular beds, size and function of blood vessels, and/or culture conditions. Table 1 illustrates the regulation of arginase expression and activity in SMCs and ECs derived from various animal species in response to specific biochemical and biophysical stimuli. Rat aortic SMCs possess substantial arginase activity that is associated with the selective expression of arginase I (Durante et al., 1997, 2001; Wei et al., 2000). This contrasts with results obtained in human pulmonary artery SMCs where both isoforms are expressed (Chen et al., 2009). Several inducers of arginase have been identified in vascular SMCs. Our laboratory identified growth factors and cyclic mechanical strain as potent inducers of arginase I in rat aortic SMCs. Moreover, we showed that growth factors and hemodynamic forces stimulate the uptake of l-arginine and inhibit the expression of iNOS by vascular SMCs (Durante et al., 1996a, 1997, 2000). These coordinate actions of growth factors are synchronized to promote the proliferative capacity of SMCs by directing l-arginine metabolism from the formation of NO to l-ornithine, the first step in polyamine synthesis. Alternatively, the actions of cyclic strain are orchestrated to increase collagen synthesis by channeling l-arginine transport and metabolism to the production of l-proline. The combination of interleukin-13 (IL-13) and interleukin-4 also stimulates arginases I expression in rat aortic SMCs (Wei et al., 2000) while IL-13 induces the expression of arginase II via the IL-13 receptor α2 (IL-13Rα2) in human pulmonary artery SMCs (Cho et al., 2013). In addition, hypoxia selectively increases arginase II expression in SMCs isolated from human pulmonary arteries (Chen et al., 2009). The induction of arginase II by IL-13 or hypoxia contributes to the proliferation of pulmonary SMCs (Chen et al., 2012; Cho et al., 2013). Consistent with these *in vitro* findings, intimal hyperplasia in premenopausal human uterine arteries is paralleled by an elevation in the expression of both arginase I and II (Loyaga-Rendon et al., 2005; Marinova et al., 2008). Moreover, arginase activity positively correlates to the magnitude of intimal thickening in these blood vessels. Thus, the expression of both isoforms of arginase is regulated in a dynamic fashion in SMCs by distinct biochemical and biophysical stimuli to elicit discrete proliferative or fibrotic responses.

**Table 1:** **Regulation of cellular arginase expression and activity**.

Cell type	Species	Isoform	Inducer or activator	Reference
SMC	Rat	I	IL-13/IL-4	Wei et al. (2000)
	Rat	I	TGF-β1	Durante et al. (2001)
	Rat	I	Cyclic strain	Durante et al. (2000)
SMC	Human	II	IL-13	Cho et al. (2013)
	Human	II	Hypoxia	Chen et al. (2012)
EC	Rat	I and II	LPS	Buga et al. (1996)
EC	Human	I and II	LPS and TNFα	Bachetti et al. (2004)
EC	Cow	I and II	LPS and TNFα	Chicoine et al. (2004)
EC	Mouse	I	TNFα	Gao et al. (2007)
EC	Human	II	Hypoxia	Toby et al. (2010)
	Human	II	Hypoxia	Krotova et al. (2010)
	Human	II	Thrombin	Ming et al. (2004)
	Human	II	Thrombin, TRAP	Yang et al. (2006)
EC	Rat	I	Thrombin	Zhu et al. (2010)
EC	Cow	I	Hydrogen peroxide	Chandra et al. (2012)
	Cow	I	Angiotensin II, peroxynitrite	Shatanawi et al. (2011)
EC	Mouse	II	Oxidized LDL	Ryoo et al. (2011)
EC	Pig	I	Oxidized LDL	Wang et al. (2011b)
EC	Rat, human	I	Hyperglycemia, high glucose	Romero et al. (2008)
EC	Mouse	I	Hyperglycemia	Yao et al. (2013)
EC	Pig	II	Shear stress	Thacher et al. (2010)
EC	Human	I	Nitric oxide	Santhanam et al. (2007)
EC	Pig	I and II	Uric acid	Zharikov et al. (2008)

*SMC, smooth muscle cells; EC, endothelial cells; IL-13, interleukin-13; IL-4, interleukin-4; LPS, lipopolysaccharide; TGF-β1, transforming growth factor-β1; TRAP, thrombin receptor activating peptide; oxidized LDL, oxidized low-density lipoprotein*.

Endothelial dysfunction arising from impaired NO synthesis is a fundamental feature in many cardiovascular disorders. Consideration of the enzyme kinetics for NOS and arginase indicate that arginase effectively competes with NOS for l-arginine under physiologic conditions (Wu and Morris, 1998), providing a framework by which arginase can provoke endothelial malfunction. Indeed, arginase has been linked to endothelial dysfunction in an expanding number of vascular pathologies, including atherosclerosis, hypertension, uremia, aging, diabetes, ischemia-reperfusion, and hemorrhagic shock (see Durante et al., 2007; Johnson et al., 2010; Michell et al., 2011). Interestingly, a large number of factors that trigger endothelial dysfunction are able to stimulate endothelial arginase activity and/or expression. Several studies have reported that inflammatory mediators stimulate the expression of both arginase I and II, and this may be linked to the activation of the Src family tyrosine kinases in pulmonary ECs (Buga et al., 1996; Bachetti et al., 2004; Chicoine et al., 2004; Nelin et al., 2005; Gao et al., 2007; Chang et al., 2008). Hypoxia is also a potent inducer of arginase II in pulmonary ECs (Krotova et al., 2010; Toby et al., 2010). The induction of arginase II by hypoxia is likely mediated by hypoxia-inducible factor 2 since silencing this transcription factor negates the rise in arginase II expression. Interestingly, the serine protease thrombin stimulates endothelial arginase activity via at least two distinct mechanisms (Ming et al., 2004; Yang et al., 2006; Zhu et al., 2010). While thrombin-mediated increases in arginase II activity occurs via a Rho pathway, the induction of arginase I gene expression by thrombin is mediated through activator protein-1 activation (Ming et al., 2004; Zhu et al., 2010). The Rho pathway appears to play a central role in mediating the induction of endothelial arginase expression in response to many atherogenic stimuli, including angiotensin II, oxidized low-density lipoprotein, hyperglycemia, hydrogen peroxide, and peroxynitrite (Thengchaisri et al., 2006; Ryoo et al., 2011; Shatanawi et al., 2011; Wang et al., 2011b; Chandra et al., 2012; Yao et al., 2013). As observed in SMCs, hemodynamic forces also regulate arginase expression in ECs. Exposure of cultured ECs or isolated carotid arteries to unidirectional shear stress modestly elevates arginase II expression whereas oscillatory shear stress, a hemodynamic pattern known to favor plaque development, strongly induces the expression of arginase II, demonstrating that endothelial arginase expression is highly sensitive to disturbances in fluid flow (Thacher et al., 2010). Post-translational modes of regulating arginase have also been reported. In particular, NO release by iNOS stimulates arginase I activity in ECs by *S*-nitrosylating a specific cysteine residue of the protein (Santhanam et al., 2007). This nitrosylation event stabilizes the arginase I trimer and causes a sixfold increase in the affinity of the enzyme for l-arginine, allowing it to better compete with NOS for l-arginine. A direct interaction between the oxygenase domain of iNOS and arginase I was required for the nitrosylation of arginase I to occur (Dunn et al., 2011). Similarly, uric acid activates both arginase isoforms by enhancing their affinity for l-arginine but the underlying mechanism remains unresolved (Zharikov et al., 2008). Thus, numerous pathologic stimuli that cause endothelial dysfunction stimulate the expression and/or activity of arginase in ECs via multiple pathways.

## Role of Arginase in Vascular Remodeling

### Arginase in arterial injury

Considerable evidence indicates that arginase plays an integral role in neointima formation. Intimal lesions following endothelial denudation of carotid arteries are larger in diabetic rabbits relative to normoglycemic animals and they exhibit greater arginase activity and expression (Ishizaka et al., 2007). In addition, a combined transcriptomic and proteomic study identified an increase in arginase I expression in arteriotomy-injured rat carotid arteries (Forte et al., 2008). Consistent with this investigation, we recently reported that balloon injury of rat carotid arteries results in a pronounced increase in arginase I protein expression that is coupled to a sustained increase in arginase activity (Peyton et al., 2009). Arginase I expression is detected throughout the injured blood vessel but it is especially prominent in the neointima. Although arterial injury also induces iNOS synthase expression (Yan et al., 1996; Tulis et al., 2000), the concomitant elevation in arginase I compromises NO synthesis at the site of injury (Alef et al., 2011). The underlying mechanism responsible for inducing arginase I expression is not known; however, the generation of growth factors and/or inflammatory cytokines following arterial injury may be involved.

Balloon injury of rat carotid arteries results in the development of a concentric SMC-rich neointima. However, local perivascular application of the arginase inhibitors *S*-(2-boronoethyl)-l-cysteine or hydroxy-nor-l-arginine immediately after arterial injury markedly diminishes neointima formation without affecting vessel caliber (Peyton et al., 2009). The inhibition of intimal hyperplasia is independent of any increase in apoptosis but is associated with a significant decline in medial and neointimal DNA synthesis, suggesting that arginase promotes intimal thickening by stimulating the proliferation of vascular SMCs. Indeed, transfection of cultured vascular SMCs with arginase I stimulates cell growth by increasing the production of polyamines while pharmacological inhibition of arginase suppresses polyamine synthesis and SMC replication (Wei et al., 2001). We also demonstrated that arginase promotes the entry of vascular SMCs into the cell cycle since blocking arginase activity or silencing arginase I expression arrests cells in the G_0_/G_1_ phase of the cell cycle (Peyton et al., 2009). The cell cycle arrest and blockade of neointimal thickening following arginase inhibition is associated with a significant increase in the expression of the cyclin-dependent kinase inhibitor p21, a known mediator of G1 arrest. The ability of arginase to suppress polyamine synthesis may contribute to the upregulation of p21 since polyamines have been demonstrated to repress p21 gene transcription (Liu et al., 2006). In addition, the discovery that arginase stimulates collagen synthesis by SMCs may further exacerbate intimal thickening by increasing collagen deposition in injured blood vessels (Durante et al., 2000, 2001). The proliferative and fibrotic actions of arginase I are further amplified by the capacity of arginase I to compete with iNOS for l-arginine and restrict the generation of NO, which is an established inhibitor of SMC proliferation and collagen synthesis (Garg and Hassid, 1989; Kolpakov et al., 1995).

### Arginase in pulmonary and arterial hypertension

Vascular remodeling is a seminal feature in pulmonary arterial hypertension (PAH) that leads to increased pulmonary vascular resistance and reduced compliance. It is characterized by the pronounced thickening of blood vessels and marked by increases in the proliferation of pulmonary artery SMCs and ECs, the extension of SMCs into smaller, non-muscular pulmonary arteries within the respiratory sinus (neovascularization), and enhanced deposition of extracellular matrix, including collagen (Humbert et al., 2004). EC dysfunction is also observed in PAH and this can further compromise pulmonary blood flow and lead to thrombosis. Recent work suggests that arginase contributes to vascular remodeling in PAH. Both isoforms of arginase are expressed in the lungs of mice exposed to chronic hypoxia but only elevated levels of arginase II are detected in pulmonary arterial ECs of patients with PAH (Xu et al., 2004; Jin et al., 2010). The induction of arginase activity in the pulmonary circulation is paralleled by the development of EC dysfunction and decreases in NO synthesis, suggesting competition between arginase and eNOS for l-arginine (Xu et al., 2004; Sasaki et al., 2007). Increased expression of arginase II is also observed in the pulmonary vasculature of a novel genetic model of PAH in which IL-13 is specifically overexpressed in the lung (Cho et al., 2013). These transgenic mice spontaneously develop PAH with obvious vascular remodeling exemplified by lung fibrosis, prominent medial thickening of pulmonary arteries, and neovascularization of small pulmonary arteries. While the expression of both arginase isoforms is found in alveolar macrophages, only arginase II expression is noted in vascular SMCs and ECs. Interestingly, deletion of arginase II decreases medial wall thickening of pulmonary arteries and reduces the frequency of neovascularization of small pulmonary arteries in IL-13 overexpressing transgenic mice, demonstrating that arginase II contributes to pathologic vascular remodeling in these animals.

Arginase II drives arterial thickening in PAH by stimulating the proliferation of vascular SMCs. Treatment of human pulmonary artery SMCs with IL-13 induces the expression of arginase II and this is associated with a marked increase in cell growth (Cho et al., 2013). Moreover, knocking down arginase II blocks the IL-13-mediated increase in SMC proliferation. Similarly, pulmonary SMCs exposed to hypoxia exhibit increases in arginase II mRNA and protein expression (Chen et al., 2009). Arginase inhibition with *S*-(2-boronoethyl)-l-cysteine or molecular silencing of arginase II expression completely prevents hypoxia-induced SMC proliferation. Since enhanced endothelial arginase II expression is associated with prominent intimal thickening in human PAH patients (Xu et al., 2004), arginase II may also contribute to the growth of ECs in this disorder. In support of this proposal, transfection of bovine coronary ECs with arginase II increases cell proliferation in a manner that is strictly dependent on polyamine synthesis (Li et al., 2002). In addition, hypoxia-induced proliferation of human pulmonary microvascular ECs is blocked by arginase inhibition (Toby et al., 2010). Thus, the ability of arginase II to stimulate the proliferation of both SMCs and ECs may play an essential role in propelling the obstructive remodeling response observed in PAH. Furthermore, arginase-mediated increases in collagen accumulation may contribute to the development of pulmonary arterial stiffness and fibrosis in PAH (Kobs and Chesler, 2006).

Arginase also influences vascular remodeling in arterial hypertension. Arginase activity is elevated in a number of animal models of essential or secondary hypertension. We previously reported that skeletal muscle arterioles from salt-loaded, salt-sensitive hypertensive rats express higher levels of arginase I and II, and that endothelial dysfunction in this vascular bed is corrected by arginase inhibition (Johnson et al., 2005). Restoration of EC function following arginase inhibition has also been described in deoxycorticosterone acetate-salt hypertensive rats, renovascular hypertensive pigs, and spontaneously hypertensive rats (SHR) (Rodriguez et al., 2000; Zhang et al., 2004; Demougeot et al., 2005; Johnson et al., 2005). In addition, upregulation of arginase activity contributes to attenuation of cutaneous vasodilation in hypertensive patients (Holowatz and Kenney, 2007). Aside from improving endothelial function, arginase inhibitors prevent the development of hypertension when given to pre-hypertensive or young adult SHR (Bagnost et al., 2008). More recently, chronic pharmacological inhibition of arginase was also found to sustainably reduce blood pressure in fully developed hypertensive SHR (Bagnost et al., 2010). Notably, arginase inhibition prevents remodeling of the aorta in these animals. There is a significant decline in aortic medial wall thickness, aortic media to lumen ratio, and type I collagen content in SHR treated with hydroxy-nor-l-arginine. In addition, arginase inhibition dramatically increases the arterial compliance of carotid arteries in SHR. However, arginase inhibition has no effect on the remodeling of mesenteric arteries, suggesting that arginase-mediated vascular remodeling is vessel dependent in these animals. The ability of arginase inhibition to repress aortic remodeling and arterial stiffness likely occurs due to decreases in SMC proliferation and collagen synthesis, and improvements in EC function. Interestingly, arginase I is selectively induced in the vasculature of SHRs suggesting that arginase I, rather than arginase II, mediates hypertensive vascular remodeling in conduit arteries of the systemic circulation.

### Arginase in aging

Aging is associated with changes in arterial wall structure and function. The most frequent modifications are luminal enlargement, vessel wall thickening due to intimal and medial expansion, elastin depletion and fragmentation, collagen and calcium deposition, glycation of proteins, and impaired vasomotor function associated with endothelial dysfunction (Virmani et al., 1991; Taddei et al., 2001; Mirea et al., 2012). These structural and functional alterations in aging contribute to increased vascular stiffness, which is an independent risk factor for cardiovascular morbidity and mortality (Sutton-Tyrrell et al., 2005; Dolan et al., 2006; Mattace-Raso et al., 2006). Accumulating evidence indicates that arginase contributes to aging-associated EC dysfunction and arterial stiffening. In aged rats, the upregulation of iNOS activity in blood vessels induces the *S*-nitrosylation and activation of arginase I (Santhanam et al., 2007). Although arginase-mediated depletion of l-arginine reduces NO synthesis, the tight physical coupling between iNOS and arginase I is likely sufficient to sustain *S*-nitrosylation and activation of arginase I (Dunn et al., 2011). Importantly, arginase inhibition restores NO synthesis and reverses endothelial dysfunction and vascular stiffness in old rats (Kim et al., 2009). Recent findings also support a role for arginase in mediating endothelial dysfunction in the circulation of aged human skin (Holowatz et al., 2006). Interestingly, increased arginase II activity contributes to endothelial dysfunction in aged mice, indicating that distinct isoforms of arginase are activated and provoke EC dysfunction in different animal species of aging (Shin et al., 2012).

### Arginase in atherosclerosis

Emerging evidence indicates that arginase also contributes to the development of atherosclerotic lesions. Endothelial arginase II activity is significantly increased in apolipoprotein E (apoE)-deficient hypercholesterolemic mice or in wild-type animals fed a high cholesterol diet in the absence of any increase in arginase II expression (Ryoo et al., 2008). Strikingly, pharmacological blockade of arginase reduces plaque burden by approximately 50% and markedly reduces average wall thickness of the ascending aorta in apoE-deficient animals. In addition, arginase inhibition improves arterial compliance in apoE-null mice to levels seen in wild-type animals. Similarly, deletion of arginase II in apoE-deficient mice fed either a high fat or high cholesterol diet results in smaller arterial lesions. In addition, the size of necrotic cores in advanced lesions is substantially reduced in arginase II-apoE double knockout mice despite comparable levels of circulating cholesterol and triglycerides (Ming et al., 2012). In contrast, apoE-deficient transgenic mice with EC-specific overexpression of arginase II exhibit increased aortic lesion development without a change in plasma lipids (Vaisman et al., 2012). Together, these animal studies demonstrate that elevations in endothelial arginase II activity or expression contributes to arterial stiffness and the development of atherosclerotic lesions that possess a vulnerable phenotype, independent of any alteration in the lipid profile.

There are several potential mechanisms by which arginase II exerts its atherogenic effect. Since impaired endothelial dysfunction and NO release plays an important role in the development and progression of atherosclerosis (Lerman and Zeiher, 2005), the ability of arginase to evoke endothelial dysfunction is highly significant. Endothelial function and NO synthesis are compromised in apoE-deficient mice but arginase inhibition or arginase II gene deletion restores NO production and endothelial function in these animals (Ryoo et al., 2008). Importantly, arginase inhibition has recently been demonstrated to acutely improve endothelial function in patients with coronary artery disease (Shemyakin et al., 2012). The beneficial effect of arginase inhibitors in these subjects is completely dependent on the increased bioavailability of NO. In-line with these findings, endothelial overexpression of arginase II induces endothelial dysfunction and hypertension in mice, further underscoring the detrimental nature of this enzyme in the cardiovascular system (Vaisman et al., 2012).

Arginase II may also promote atherogenesis by augmenting the inflammatory response of leukocytes. Monocytes and macrophages are key cellular protagonists of atherosclerosis that play a fundamental role in the initiation, progression, and rupture of atherosclerotic plaques (Libby, 2002; Mantovani et al., 2009; Shibata and Glass, 2009). Monocytes readily infiltrate vascular lesions, differentiate into macrophages, ingest lipoprotein particles, and give rise to foam cells. Macrophages are major cellular contributors to the lesion’s physical bulk and contribute to the evolution of the plaque by secreting inflammatory cytokines and reactive oxygen species. They also weaken and destabilize plaque by releasing various proteases. However, monocytes and macrophages are phenotypically diverse and can express pro- and anti-atherogenic programs. Interestingly, recent work suggests that arginase influences the polarization of these cells. In particular, the expression of arginase II is coupled with the classically activated M1 macrophage phenotype, which fosters the release of inflammatory mediators and proteases, and is associated with advanced atherosclerotic lesions (Khallou-Laschet et al., 2010; Ming et al., 2012). In addition, silencing arginase II expression in human monocytes suppresses their pro-inflammatory function: both monocyte adhesion onto activated ECs and inflammatory cytokine production is blocked. The pro-inflammatory role of arginase II is also observed *in vivo*. Targeted disruption of arginase II blunts the infiltration of macrophages into various organs and the expression of inflammatory cytokines in adipose tissue of mice fed a high fat diet (Ming et al., 2012). Arginase II deficiency also limits macrophage content and cytokine expression in atherosclerotic plaques of apoE-null mice. Moreover, adoptive transfer experiments reveals that fewer donor arginase II-deficient monocytes than arginase II-competent macrophages infiltrate into the plaque of apoE-depleted mice, while apoE-arginase II double knockout mice accumulate fewer monocytes than do recipient single knockout apoE animals. Thus, arginase II may exacerbate arterial lesion formation by promoting both endothelial dysfunction and the pro-inflammatory potential of monocytes and macrophages.

Surprisingly, an atheroprotective role for arginase I has been identified in macrophages. Using subtractive suppression hybridization to screen for differentially expressed genes in macrophages obtained from two strains of rabbits with genetically determined high and low predisposition to atherosclerosis, arginase I is found to be expressed at higher levels in macrophages with low atherosclerotic response relative to those with a high response (Teupser et al., 2006). Consistent with these findings, a marked reduction in arginase I gene expression is noted in foam cell macrophages compared with non-foamy macrophages in cholesterol-fed rabbits (Thomas et al., 2007). Immunohistochemistry reveals arginase I expression in superficial and adventitial macrophages and by foam cells and SMCs underlying the fibrous cap with reduced expression deeper within the plaque of advanced rabbit lesions. In human carotid atherosclerotic plaques, arginase I is also widely distributed in the superficial cell layers but is absent from macrophages close to or within the lipid core. The expression of arginase I by macrophages is associated with the alternatively activated M2 macrophage which promotes resolution of inflammation by releasing anti-inflammatory cytokines and engulfing cellular debris (Martinez et al., 2009). Thus, arginase I may resolve inflammatory reactions within atherosclerotic lesions by regulating macrophage polarization. In support of this notion, mRNA expression profiling experiments found that macrophages from regressing murine plaques display enhanced levels of arginase I (Feig et al., 2012). Furthermore, arginase I expression modulates the inflammatory response of vascular SMCs. Overexpression of arginase I inhibits cytokine production and the activation of the pro-inflammatory transcription factor, nuclear factor-κB, by SMCs (Wang et al., 2011a). In addition, intraplaque gene delivery of arginase I reduces macrophage infiltration and inflammation in arterial lesions of rabbits while local silencing of arginase I expression aggravates these responses. Aside from attenuating inflammation, the expression of arginase I in SMCs near the fibrous cap of atherosclerotic lesions may also promote plaque stability by stimulating SMC proliferation.

### Model for the regulation of vascular remodeling by arginase

**Figure 2:**
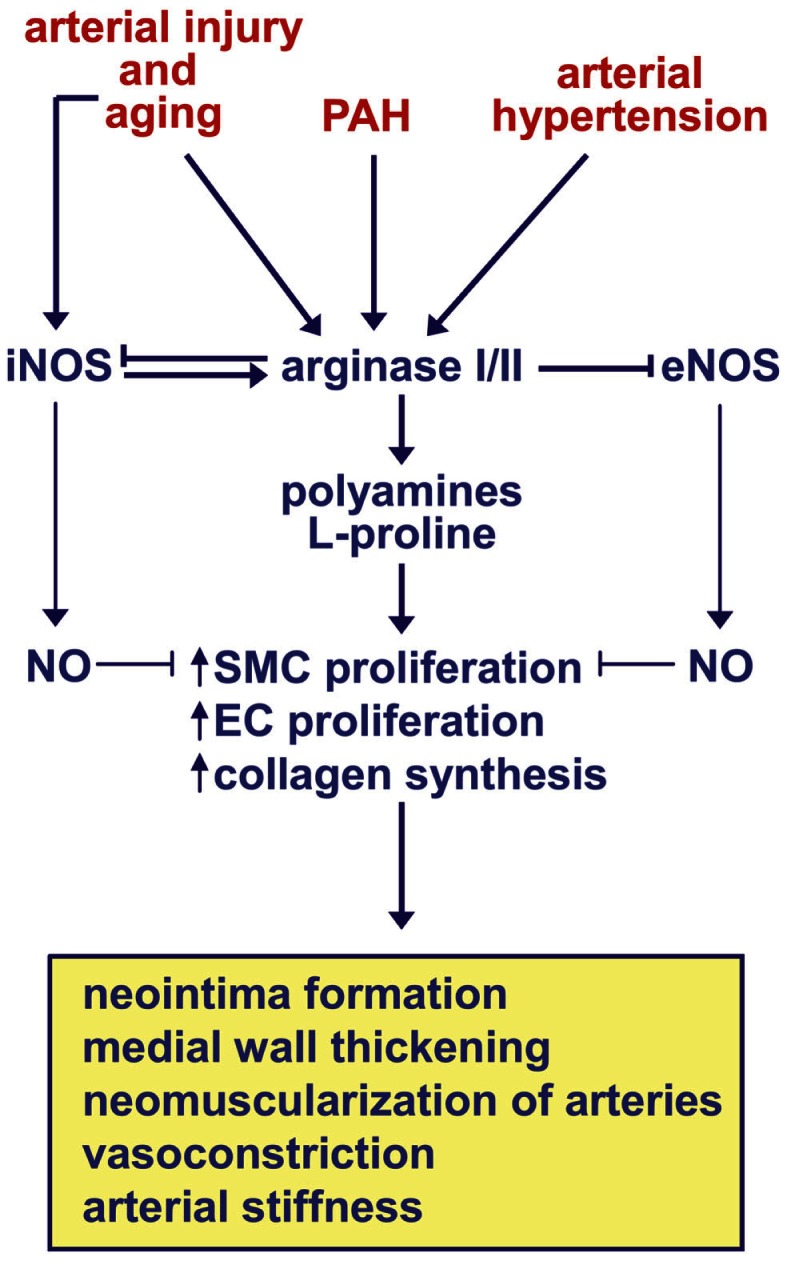
**Model for the regulation of vascular remodeling by arginase in arterial injury, pulmonary arterial hypertension (PAH), arterial hypertension, and aging**. Arterial injury, PAH, arterial hypertension, and aging stimulates the activity and/or expression of arginase I or II in blood vessels, while arterial injury and aging also stimulates the expression of inducible nitric oxide (NO) synthase (iNOS). The induction of iNOS by aging or arterial injury may further augment the activation of arginase I by nitrosylating a specific cysteine residue of the enzyme. Activation or induction of both arginase isoforms stimulate vascular smooth muscle cell (SMC) and endothelial cell (EC) proliferation, and collagen synthesis by increasing the production of polyamines and l-proline. These cellular actions of arginase are further amplified by the ability of arginase to limit the metabolism of l-arginine by iNOS and endothelial NO synthase (eNOS) to NO, which is a known inhibitor of SMC proliferation and collagen synthesis. Collectively, these arginase-driven events result in neointima formation, medial wall thickening, neovascularization of small arteries, vasoconstriction, and/or arterial stiffness.

**Figure 3:**
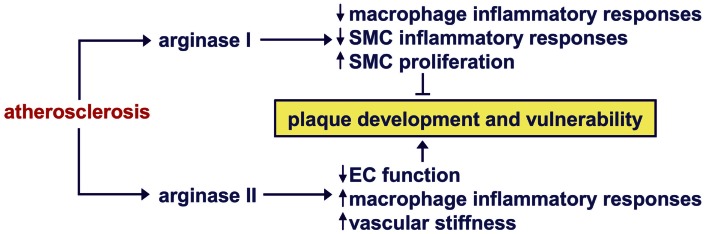
**Model for the regulation of atherosclerotic plaque progression and stability by arginase**. The induction of arginase II activity in atherosclerosis stimulates plaque development and vulnerability by stimulating endothelial cell (EC) dysfunction, the inflammatory potential of macrophages, and arterial stiffness. In contrast, the induction of arginase I expression in atherosclerosis promotes plaque stability by blocking the inflammatory responses of macrophages and vascular smooth muscle cells (SMCs) and stimulating the proliferation of vascular SMCs.

Recent studies implicate arginase as a critical contributor to the structural and functional remodeling of arteries that underlies a number of vascular diseases. Figure 2 presents a model where arginase plays a central role in vessel wall remodeling in response to arterial injury, PAH, arterial hypertension, and aging. All four of these pathologic states stimulate the activity and/or expression arginase I or II in blood vessels, while arterial injury and aging also induces the expression of iNOS. The induction of iNOS by aging or arterial injury may further augment the activation of arginase I by nitrosylating a specific cysteine residue of the enzyme. Activation and/or induction of both arginase isoforms stimulate SMC and EC proliferation, and/or collagen synthesis by increasing the production of polyamines and l-proline. These cellular actions of arginase are further amplified by the ability of arginase to limit the metabolism of l-arginine by iNOS and eNOS to NO, which is a known inhibitor of SMC proliferation and collagen synthesis. Collectively, these arginase-driven events result in neointima formation, medial wall thickening, neovascularization of small arteries, vasoconstriction, and/or arterial stiffness. Emerging work indicates that arginase also contributes to arterial remodeling in atherosclerosis; however, the role played by the two arginase isoforms appears to differ (Figure 3). The induction of arginase II activity in atherosclerosis stimulates plaque development and vulnerability, and vascular stiffness by triggering EC dysfunction and the inflammatory potential of macrophages. In contrast, the induction of arginase I expression promotes plaque stability by blocking the inflammatory responses of macrophages and SMCs, and by stimulating the proliferation of vascular SMCs.

## Therapeutic Strategies Targeting Arginase in Vascular Remodeling

Several approaches can be employed to target arginase in vascular remodeling. One promising approach involves the development and use of pharmacological inhibitors. While early attempts to establish a role for arginase were hampered by the lack of potent and specific inhibitors of arginase, recent development of boronic acid and N-hydroxy-guanidinium derivatives, such as 2(*S*)-amino-6-boronohexanoic acid, *S*-(2-boronoethyl)-l-cysteine and *N*^G^-hydroxy-nor-l-arginine, has yielded highly potent competitive inhibitors that can readily be used to probe arginase function (Christianson, 2005). The effectiveness of these arginase inhibitors has been demonstrated in various *in vitro* and *in vivo* models, including humans (see Durante et al., 2007; Holowatz and Kenney, 2007; Morris, 2009; Shemyakin et al., 2012). However, further pharmacokinetic and toxicology studies are needed in order to optimize safe and effective therapeutic regimens for these inhibitors. One important limitation with currently available pharmacological inhibitors is their inability to provide isoform-selective inhibition of arginase. The development of small molecule inhibitors that discriminate between arginase I and II will be critical when targeting vascular disorders in which different arginase isoforms elicit disparate actions in blood vessels. Some concern has also been raised over possible non-specific actions of certain arginase inhibitors (Huynh et al., 2009). Given the paucity of isoform-selective arginase inhibitors, small interference RNA (siRNA) has been extensively employed to silence arginase I and II expression in cultured vascular cells. In addition, siRNA has been successfully used to knock down arginase expression in blood vessels both *ex vivo* and *in vivo* (Wang et al., 2011a; Shin et al., 2012). Although siRNA technology holds great promise, current difficulties in delivery, and potential safety and off-target effects limit the clinical efficacy of this approach (Keaney et al., 2011).

Another potential strategy in blocking arginase activity involves the use of dietary antioxidants. Cocoa flavanols lower arginase II mRNA expression and activity in cultured human ECs while oral ingestion of flavanols decreases arginase activity in rat kidney and in human erythrocytes (Schnorr et al., 2008). The catechin, epicatechin gallate, improves scar formation during incisional wound healing in rats and this is associated with a decrease in arginase I expression and activity (Kapoor et al., 2004). In addition, the intake of red wine polyphenols ameliorates endothelial dysfunction and arginase I expression in blood vessels of middle-aged rats (Dal-Ros et al., 2012). Furthermore, the bioflavonoid, quercetin, suppresses liver arginase activity in acute renal failure (Nikolic et al., 2003). Interestingly, Danshen, a traditional Chinese herbal medicine that is commonly used for the prevention and treatment of cardiovascular disease, may exert its beneficial vascular effects, in part, through the inhibition of arginase (Joe et al., 2012). Consequently, a variety of dietary approaches may be used in limiting arginase activation; however, detailed clinical studies are needed to establish the efficacy of any nutritional approach.

There is a growing awareness that clinically relevant drugs are able to suppress arginase activity. This is best exemplified by the statin family of drugs which lower circulating cholesterol levels by inhibiting the enzyme 3-hydroxy-3-methyl-glutaryl-CoA reductase. Several statins are capable of inhibiting arginase activity. Daily treatment of diabetic rats with simvastatin blunts diabetes-induced endothelial dysfunction and arginase I expression (Romero et al., 2008). In addition, lovastatin and simvastatin completely block the induction of arginase II activity by oxidized low-density lipoprotein in human ECs (Ryoo et al., 2011). Furthermore, in an acute murine model of allergic asthma, simvastatin represses arginase I protein expression and early hallmarks of airway remodeling (Zeki et al., 2010). Moreover, oral atorvastatin therapy restores cutaneous microvascular function by decreasing arginase activity in hypercholesterolemic humans (Holowatz et al., 2011), indicating that arginase inhibition contributes to the pleiotropic and anti-atherogenic actions of statins. The mechanism by which statins reduce arginase activity likely occurs through inhibition of RhoA and Rho-kinase signaling (Ryoo et al., 2011). Aside from statins, the angiotensin-converting enzyme inhibitor, lisinopril, reverses the elevation in arginase activity in erythrocytes from patients with atherosclerosis and hypertension while the phosphodiesterase type 3 inhibitor, cilostamide, inhibits hypoxia-induced arginase II expression in human pulmonary artery SMCs (Chen et al., 2012; Kosenko et al., 2012). Furthermore, oral administration of 17β-estradiol in oophorectomized rabbits fed a cholesterol enriched diet decreases atheromatous lesions and this is accompanied by reductions in both arginase I and II expression, illustrating a potential hormonal approach in targeting arginase (Hayashi et al., 2006). Thus, an increasing number of drugs used in the treatment of cardiovascular disease have been shown to inhibit arginase expression and/or activity, and some evidence suggests that arginase inhibition contributes to their therapeutic efficacy.

## Conclusion

Recent experimental studies have implicated arginase as a key contributor to the detrimental vascular remodeling response observed in arterial injury, pulmonary and arterial hypertension, aging, and atherosclerosis. The application of potent pharmacological inhibitors of arginase has provided important novel insight into the role of arginase in regulating vascular cell function. In addition, they have proven effective in improving vascular remodeling in animal models of arterial disease and represent an attractive near-term clinical strategy. Interestingly, several natural occurring antioxidants have been demonstrated to block arginase activity and/or expression, providing a potential dietary approach in targeting the enzyme. Given the differential expression of arginase I and II between discrete vascular cells and blood vessels, and their divergent actions in certain pathological settings, the development of potent isoform-selective arginase inhibitors is highly desirable. While molecular approaches using siRNA to silence arginase expression are promising, further refinements in this technology are needed to permit the clinical targeting of distinct arginase isoforms. Future translational studies will determine the success any of these strategies in treating or preventing obstructive fibroproliferative vascular disease.

## Conflict of Interest Statement

The authors declare that the research was conducted in the absence of any commercial or financial relationships that could be construed as a potential conflict of interest.
